# First person – Jasmine Alqassar

**DOI:** 10.1242/bio.062364

**Published:** 2025-11-28

## Abstract

First Person is a series of interviews with the first authors of a selection of papers published in Biology Open, helping researchers promote themselves alongside their papers. Jasmine Alqassar is first author on ‘ WntA expression and wing transcriptomics illuminate the evolution of stripe patterns in skipper butterflies’, published in BiO. Jasmine is a PhD student in the lab of Arnaud Martin at The George Washington University, Washington, USA, investigating how evolutionary changes in developmental processes facilitate adaptation.


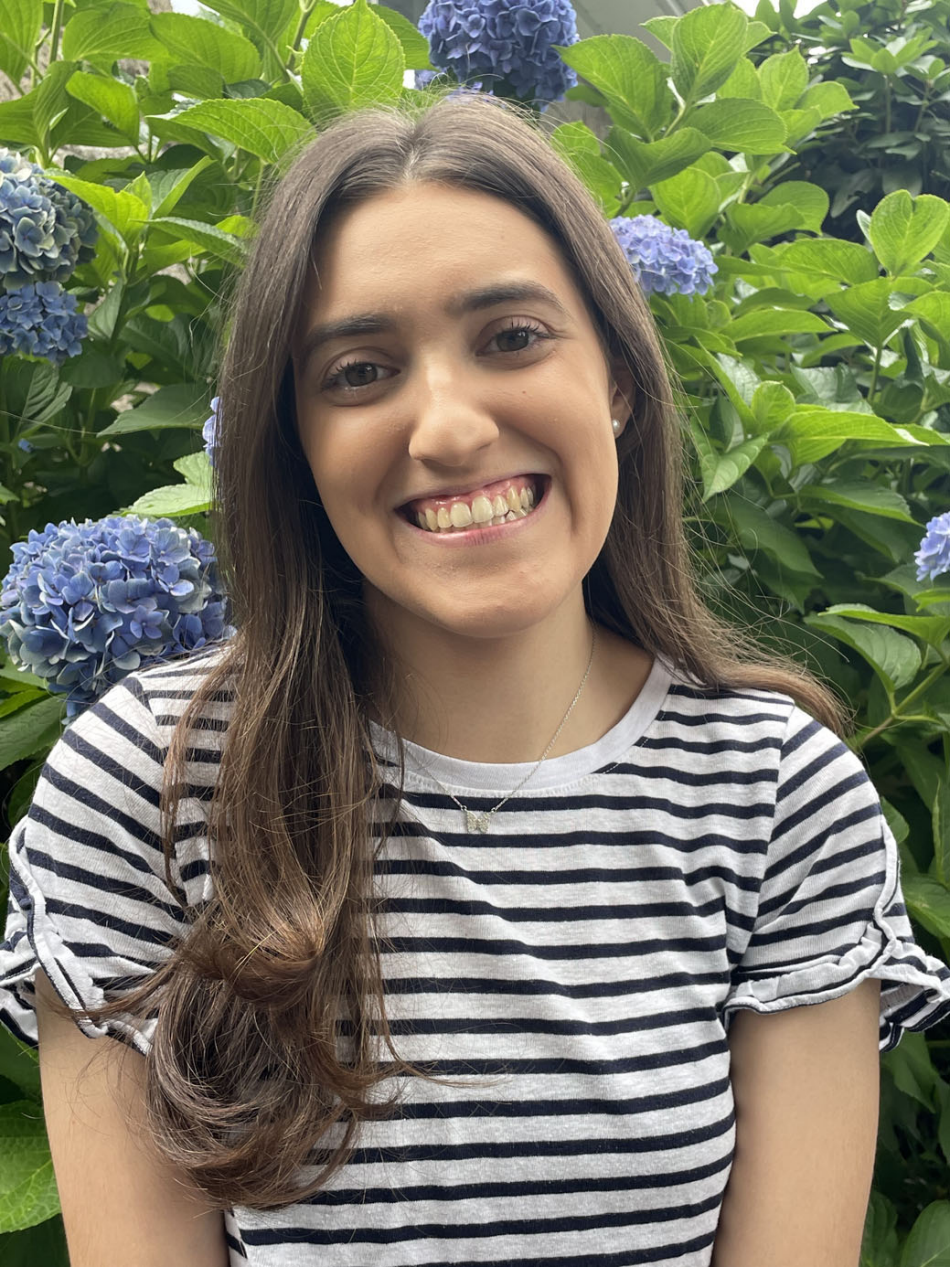


**Jasmine Alqassar**

**Describe your scientific journey and your current research focus**

My scientific journey started at Boston University, where as an undergraduate I studied cell biology, molecular biology, and genetics. During this time, I took an evolutionary biology course with Dr Sean Mullen, which led me to become fascinated with the ability of small genetic changes to facilitate adaptation. I joined Dr Mullen's lab as an undergraduate and ultimately continued as a Master's student through the joint BA/MS program, to study the webbing clothes moth, *Tineola bisselliella*, and its fascinating ability to digest keratin. As a Master's student, I used bioinformatics to explore the genomic basis for this potentially adaptive trait. My Master's made me realise I wanted to continue to work on the genomics of butterflies and moths in the future using both bioinformatics techniques as well as functional tests, such as CRISPR, transgenesis, and tissue staining to understand the genetic changes underlying evolution. This led me to the field of evolutionary developmental biology (evo-devo) and the lab of Dr Arnaud Martin at The George Washington University where I am currently studying the evo-devo of silk production in the silk glands of pantry moths, *Plodia interpunctella*.

**Who or what inspired you to become a scientist?**

I was inspired to become a scientist by my sixth-grade science teacher, Mrs Katharine Bowers. She saw my passion and curiosity for science and encouraged me to join the after-school Science Olympiad club she advised. She helped me to explore the different areas of science, and ultimately when I decided I wanted to pursue a scientific career in genetics, explained to me how I could achieve this goal. Her mentorship set me on the path to become the researcher I am today.

…the gene *WntA* is a crucial marker of stripe elements early in development, confirming the gene's function has been conserved over long evolutionary distances…

**How would you explain the main finding of your paper?**

The genetic basis for the stripe elements of butterfly wings has been found in the Nymphalidae butterfly family to be patterned early in wing development by expression of the gene *WntA*. In this paper, we aimed to explore if *WntA* has maintained this patterning role of stripe elements over 95 million years of evolution, in the Hesperiidae family of skipper butterflies. Our paper used RNA sequencing and mRNA tissue staining methods for the gene *WntA* in the silver-spotted skipper, *Epargyreus clarus*, to uncover the patterning of stripe elements in the Hesperiidae family. We found that the gene *WntA* is a crucial marker of stripe elements early in development, confirming the gene's function has been conserved over long evolutionary distances and uncovered other candidate genes potentially involved in the early patterning of wings.

**What are the potential implications of this finding for your field of research?**

Our findings expand the knowledge of butterfly wing patterning and confirm the conserved role of *WntA* in stripe patterning. Additionally, our RNA sequencing results uncovered new potential candidates involved in early patterning of butterfly wings.

**Figure:**
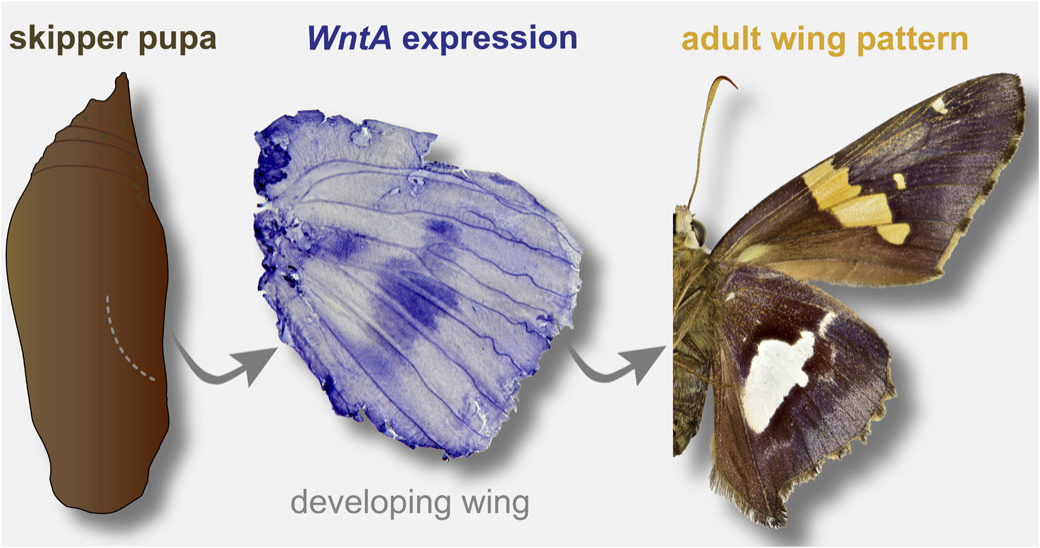
*WntA* expression in the developing butterfly wing marks the future stripe pattern elements in butterfly wings.

**Which part of this research project was the most rewarding?**

This research project started in 2017, well before my time in the lab, with many people working on the project throughout the years. When I joined the lab, I was given the opportunity to finish the project as I had already developed the bioinformatics skills needed to finish the analysis of RNA sequencing data, and it would allow me to learn new techniques in the lab, such as how to dissect a pupal butterfly wing and perform colorimetric *in situ* hybridization experiments to stain for mRNA. It was incredibly rewarding to be able to finish such a long-standing collaborative project and I really enjoyed getting to dive into the science behind butterfly wing pattern development.

**What do you enjoy most about being an early-career researcher?**

I really enjoy the freedom of exploration allowed to me as an early-career researcher. My advisor, Dr Arnaud Martin, is incredibly supportive of my exploration of different projects and topics as well as opportunities to expand my training. This paper is a great example of this, as I had not previously worked on butterfly wing patterning, so this project allowed me to explore a whole different field of research and techniques.

**What piece of advice would you give to the next generation of researchers?**

My advice is to lead with curiosity in your science. Your research won't always follow the original plan you had, so stay open to unexpected findings, as they often lead to the most exciting discoveries.

**What's next for you?**

Next, I will be diving back into silk gland research to explore how transcription factors specify different compartments of gene expression within pantry moth silk glands.
